# A report on the blended short-term supplementary course on “Developmental Care for infants and toddlers” taught with a multidisciplinary approach for pediatricians -qualitative and quantitative study

**DOI:** 10.1186/s12909-022-03943-1

**Published:** 2022-12-17

**Authors:** Seifollah Heidarabadi, Mohammad Barzegar, Hakimeh Hazrati, Hassan Shahrokhi, Shahrooz Nemati, Nahideh Hasani Khiabani, Zahra Maleki

**Affiliations:** 1 Pediatric Health Research Center, Tabriz University of Medical Sciences, Tabriz, Iran; 2 Medical Education Research Center, Health Management and Safety Promotion Research Institute, Tabriz University of Medical Sciences, Tabriz, Iran; 3 Research Center of Psychiatry and Behavioral Sciences, Hassan Shahrokhi, Tabriz University of Medical Sciences, Tabriz, Iran; 4 Educational Department, Shahrooz Nemati, University of Tabriz, Tabriz, Iran; 5 Tabriz, Iran

**Keywords:** Pediatric, Multidisciplinary, Education, Qualitative and quantitative study

## Abstract

**Background:**

The implementation of follow-up programs for high-risk infants and toddlers aimed to promptly diagnose developmental delays and disorders and initiate early intervention to help improve their developmental status, reduce their care costs in the future, as well as improve their productivity as members of society. There is a lack of qualified specialists in the infant and toddler development field in Iran. To compensate for the lack of training in this area, for the first time, Tabriz University of Medical Sciences has designed a short-term supplementary course of “Developmental Care for Infants and Toddlers”. Due to the multidisciplinary nature of the field of child development, this course has been designed as such. The current study aimed to evaluate this course and explain the graduates’ relevant experiences using a multidisciplinary approach.

**Methods:**

The current study is a quantitative/qualitative study conducted in two phases. In the first step, the learners were asked to assess the teaching quality of the short-term supplementary course of “Developmental Care for Infants and Toddlers” in 5 areas of “educational design,“ “course administrators’ support”, " learners’ motivation “, “acquisition of general learning and specialized skills” using the Australian Course Experience Questionnaire (CEQ). All graduates completed the questionnaires. The data from the questionnaires were analyzed using descriptive statistics of medians, and interquartile ranges in SPSS software. The second step was a qualitative study to explain the graduates’ experiences of this course with a multidisciplinary approach. The samples were selected using a purposive sampling technique. The samples were those who had completed the course mentioned above, had rich experiences in this field, and were willing to share them. The data were collected through semi-structured interviews and analyzed using conventional content analysis.

**Results:**

In general, the graduates’ satisfaction with the course in terms of the five areas studied was evaluated as follows: Educational design, motivating participants to do their best had the greatest median. 5(4-5), Learning objectives; in all items, the median was 4. Course content and resources; all items median were 4 .Relevant learner assessment methods; in all items, the median was 4.The median learners’ satisfaction in the areas of “course administrators’ support” in all items was 4 and “learners’ motivation “, was 5. Learner’s motivation” in all items it was 4, indicating the highest level of satisfaction with the “acquisition of specialized skills”. In the area of education design, the highest satisfaction was found with the appropriateness of teaching strategies. The codes extracted from the analysis of interviews with the graduates, are divided into four categories: “Ethical and professional commitment of course teachers”, “Being a role model in the observance of patients and their caregivers’ rights”, “Course planning with a multidisciplinary approach and teamwork” as well as “The use of virtual platforms to strengthen and maintain team communication between learners”.

**Conclusion:**

This course was the first experience of applying a multidisciplinary approach in an interprofessional course. Ideally, it is indispensable for the Iranian medical education system to move away from specialization and connect all related specialties and disciplines to achieve its educational and therapeutic goals. Therefore, the design of this course can be applied as an educational model for other disciplines and clinical courses.

## Background

Recent decades have witnessed remarkable advances in the care and treatment of high-risk infants worldwide. According to global studies, a significant proportion of children (13–17%) suffers from developmental disorders[1], and merely 16–18% of children with speech disorders, mental retardation, learning disabilities, and behavioral disorders are identified before they begin school[2, 3]. Moreover, various national and international studies have indicated that the implementation of follow-up programs for high-risk infants and toddlers that promptly diagnose developmental delays and disorders and initiate early intervention will help reduce complications and prevent the continuation of developmental disorders, save time and cost, reduce care costs in the future, and increase their productivity as members of society [4, 5]. UNICEF, and WHO underscore the importance of the “Care for Child Development” program [6]. Furthermore, in recent years efforts have been made in Iran to develop the neonatal intensive care unit (NICU) to provide care to critically ill infants by establishing a committee for newborns and mothers, as well as taking other measures.

Monitoring high-risk infants facilitates the diagnosis of potential abnormalities physical and developmental disorders as well as prompt intervention., In addition, it guides the lower levels of the healthcare system to help them provide better care for these infants. Given the fact that such follow-up and intervention are not thoroughly explored and accurately known in the Iranian health system as measures currently taken in the field of pediatrics and its subfields such as pediatric neurology and psychiatry or various rehabilitation-related fields, as well as the lack of qualified specialists in the field of infant and toddler development, the Child Health Office (CHO) of the Ministry of Health and Medical Education accentuates the establishment and operation of Child Development Centers (CDC) in all provinces. Accordingly, these centers are required to receive and manage children diagnosed with developmental disorders at risk or suspected of developmental delay during the one-year development screening. For the first time in Iran, the Tabriz University of Medical Sciences has developed, organized, and taught the short-term supplementary course on “Developmental Care for Infants and Toddlers”. In Iran Due to the multidisciplinary nature of the child development specialty, this course was designed as such. Furthermore, teachers from various disciplines involved in diagnosing, treating, and managing developmental disorders participated in the course to impart their knowledge to the learners. Since this course is the first to be offered in Iran, it would be beneficial to evaluate the participants’ knowledge, attitude, and performance to improve the quality of future courses. Therefore, teaching quality and the nature of the multidisciplinary course were evaluated using quantitative and qualitative methods. Teaching quality was evaluated by descriptive study, since the implementation of an effective multidisciplinary approach requires evaluating cultures, beliefs, and stakeholders’ values involved in the teaching and learning process using this approach. However, an effective multidisciplinary approach will succeed or fail based on the context of the society in which it is implemented. A qualitative approach seems the most effective method of identifying the underlying reasons for differences in attitudes toward multidisciplinary education in this course. The current study aimed to evaluate “Developmental Care for Infants and Toddlers” course, and examine the learners’ experiences of this multidisciplinary course.

## Methods

This course was organized and taught in 120 working days. A 10-day workshop was organized at the beginning of the course to familiarize learners with the basics of child development and common developmental disorders and teach them how to use various developmental tools. In this workshop, teachers taught how to manage diagnostic and interventional problems as well as the theoretical and practical principles of developmental interventions in various fields, including speech therapy, psychology, occupational therapy, and physiotherapy. The learners attended Tabriz Child Development Center (TCDC) during specific pre-scheduled days each month (approximately 55 of 120 days). In addition, they attended their local CDCs on the remaining days, conducted developmental evaluations of their clients, designed their diagnostic programs, and presented them via an electronic learning management system (LMS). Afterwards, the teachers and other learners shared their opinions on the issues raised, and a conclusion was reached. The current study aimed to evaluate this course and explain the graduates’ relevant experiences using a multidisciplinary approach. This study conducted in two phases. In the first phase, the quality of teaching and course " was assessed by a questionnaire, and in the second phase, the graduates’ experiences of the multidisciplinary approach were evaluated by a qualitative study.

Phase 1: In a descriptive study, all graduates were asked to complete a questionnaire to evaluate the teaching quality of the short-term supplementary course of “Developmental Care for Infants and Toddlers” in five areas of “educational design”, “course administrators’ support”, " learners’ motivation “, “acquisition of general learning and specialized skills” using the reliable and valid Australian Course Experience Questionnaire (CEQ). Questionnaires were sent to the course graduates through email. SPSS software was used to analyze the questionnaire data using descriptive statistics of medians and interquartile ranges.

Australian Course Experience Questionnaire (CEQ). is a valid and reliable questionnaire developed by Scott in 2005 for probing key elements of the university learning process, quality of teaching and courses. (Construct validity; chi-square = 3865.72, *p* < 0.001; CFI = 0.85; normed fit index = 0.84; non-normed fit index = 0.83; based on 579 degrees of freedom; RMS = 0.04).) levels of internal consistency (Cronbach’s alpha coefficients = 0.67) [7].

The Persian version of CEQ was developed in several stages. The first step was to translate the scale from English to Persian using the instrument translation framework of the World Health Organization (WHO) in four steps:

1) pre-translation (A conceptual and the closest translation of the terms by professional translators in the healthcare field), 2)the evaluation of the pre-translation step by a panel of experts (by assembling a bi-lingual expert panel, revising and evaluating the questionnaire, resolving conceptual deficiencies and non-technical issues), 3) pre-test and interview (Performing pre-tests on individuals with full mastery of the instrument to examine the scale, identify unfamiliar words and concepts, and eliminate irrelevant words in the course evaluation.), 4) final translation (comparing the scale with the Persian version and and conducting the final edition). After close translation of the CEQ, its reliability and face, content, and construct validity were also assessed in several stages. Face validity was confirmed by seeking experts’ opinions in education, research, and psychometric analysis (a panel of 8 experts). In this stage, the degree of relevance and relationship among the items, ambiguity in words/phrases, and level of difficulty, as well as the experts’ understanding of the concepts, were assessed, and their comments were applied to the questionnaire. The face validity of the items was quantitatively assessed by using the item impact method. To this end, a five-point Likert scale was considered for each item: crucial (score of 5), important [4], moderately important [3], slightly important [2], not important at all [1]. After the target group had completed the scale, the item impact formula was used to calculate the face validity. In this stage of face validity determination, items with an impact score of < 1.5 were removed., Various factors were considered to determine the validity of the content, including grammar, vocabulary, item importance, placement, time of completion, etc. Modifications were applied following the collection of experts’ comments and consultation with the research team. Quantitative content validity was assessed by using CVR and CVI., Experts in this domain were asked to assess each item on a three-point scale (*it is necessary, it is useful but not necessary, it is not necessary*) to quantitatively determine CVR and, based on Lawshe’s table, wrong items were removed. the raters were asked to assess each item based on three criteria of specificity, simplicity, and clarity on a four-point Likert scale to determine CVI [8]. Accordingly, items with a CVI < 0.79 were deemed unacceptable and removed. (Impact score = 4.12, CVI = 0.92, CVR = 0.89)

The second step was a qualitative study that aimed to probe the nature of a multidisciplinary course which was conducted using content analysis. A purposive sampling technique was used to select the samples. Samples were drawn from those who had completed the course mentioned above, had extensive experiences in this field, and were willing to share their experiences. Semi-structured interviews were used to collect the data. Interviews were conducted at Tabriz Child Development Center (TCDC). To this end, before the interview, the researcher developed an interview guide form that included general questions covering the topic and objectives of the interview. First, a general question was posed. For example, could you please share your experiences of multidisciplinary education in this course with us? Or could you please tell us one of the most essential characteristics of this course that positively affected your education experience or your continued cooperation with others? Following the interviews, exploratory questions were posed to explore the depth of the learners’ experiences. Immediately after transcribing each interview, was transcribed immediately, and the initial coding was performed. The analysis of each interview guided conducting the next interview and designing new questions to shed light on the ambiguities of the previous interview. While conducting the interview, the interviewers (H and H) attempted to maintain the relevance of the questions to the research objectives. Before conducting the interviews, participants were informed of the detailed information of the current research through an informed consent form. Furthermore, the research objectives, their voluntary participation in the study, and the confidentiality of information were explained to them. If the interviewees agreed, the interviews were tape recorded. Each interview was immediately transcribed following the completion of the interview. Once the interviews had been listened to, the transcriptions were read several times to become immersed in the interviews Afterward, open coding was performed, i.e., semantically loaded sentences were highlighted, and Each unit of analysis was assigned a code. It was found that the codes were occasionally similar to words used by the participants and in some cases, the concept latent in the sentence was regarded as the initial code. Next, the codes were divided into categories and sub-categories based on the spectrum and characteristics. In case of a conflict between two codes, the inconsistency was resolved through discussion. Credibility, confirmability, dependability, and transferability were the four criteria used to evaluate the study’s trustworthiness. For credibility the interviewer attempted to build a good rapport with the participants to gain their trust. For dependability, the codes extracted were reviewed and the participants verified their acceptability, the process of data analysis was confirmed by the research team, and consensus on the opinions. For triangulation experts in medical education and qualitative research instructors reviewed the methodology and codes extracted from the data. Moreover, though. Data immersion was achieved through prolonged engagement with the data. Research details were meticulously documented to promote the transferability of findings to provide a detailed description of the research process so that the external observer could evaluate it., Regardless of gender, the interviews with instructors of diverse universities, ages, and positions were conducted to obtain maximum variance sampling.

## Results

A total of eight pediatricians from various cities in Iran (Tabriz, Tehran, Zanjan, Qazvin, Shiraz, Khoy) participated in this course and completed it successfully.

### Results of the first step

A Course Experience Questionnaire was used to assess the learners’ satisfaction with the course in five areas as follows: educational design, motivating participants to do their best had the greatest median. 5(4-5), Learning objectives; in all items, the median was 4, except item number 3, which was a negative question, and participants could easily discern the expectation of the question.2(1.25–2.75). Course activities; more than half of the participants were satisfied with course activities. Course content and resources; all items median were 4 except question number1, library resources (3.5(3-4.6) and question number4 (unequivocal and indicator of the summary of the materials) 3(3-4) 2(1.25–2.75). Relevant learner assessment methods; in all items, the median was 4, except for question number one, which was a negative question, and teachers evaluated practical subjects rather than memorizing them 1.0(1.0–1.0). (Table 1).

**Table 1:** Learners’ satisfaction level with the educational design of the short-term supplementary course on “Developmental Care for Infants and Toddlers.“

Area	Questions	medians (quartile1, 3)
The use of teaching strategies	The teachers of this course motivated me to do	5(4–5)
	My teachers devote a significant amount of time to providing feedback on my work.	4(4–5)
	I was genuinely accommodated by the teachers when it came to an understanding any difficulties, I might be experiencing with my work	4(3–5)
	My teachers provided me with constructive feedback regarding my progress.	4(4–5)
	Teachers selected and explained exciting scenarios.	4(4–5)
	Teachers did their best to make their subjects interesting.	4(4–5)
Learning objectives	In terms of work standards, it was always unequivocal what was expected.	4(4–4)
	The course objectives and expectations were equivocally stated.	4(3–4)
	I often found it difficult to understand what was expected of me in this course.	2(1.25–2.75)
	. Students were made aware of what teachers expected of them from the beginning	4(3.25-4)
Course activities	I was overburdened with work.	3(2.25-3)
	The time provided to me for learning was generally adequate.	4(4–4)
	I was under remarkable pressure to perform well in this course.	2.5(2–3)
	It was impossible to comprehend everything in this course due to the sheer amount of work involved.	2(1.25-2)
Course content and resources	Could the library resources appropriately meet my needs?	3.5(3-4.6)
	Was the use of information technology in teaching and learning effective where it was used?	4.5(3–5)
	. Were the pertinent and appropriate learning materials available when I needed them?	4(3-4.75)
	Were the study materials unequivocal and concise?	3(3–4)
	Was the course well-designed with relevant and up-to-date materials?	4(4–4)
	How relevant were the course resources in terms of content and worksheets?	4(4–5)
Relevant learner assessment methods	The teachers seemed to be more interested in assessing what I had memorized than understood.	1.0(1.0–1.0)
	My psychomotor skills were also assessed.	4(3–4)
	My problem-solving skills were also assessed.	4(4–5)

The results indicate that the median learners’ satisfaction in the areas of “course administrators’ support” in all items was 4 and “learners’ motivation “, was 5 (Table 2).

**Table 2:** The learners’ satisfaction with the course administrators’ support and learners’ motivation

Area	Question	medians (quartile1,3)
Course administrators’ support	The required resources were readily accessible.	4(4–4)
	I could access e-resources when I needed	4(4–4)
	I was satisfied with the course and professional advice provided	4(4–5)
	Health and welfare services met my requirements.	4(3.25–4.75)
Motivation of learners	It was an intellectually stimulating course for me	5(4–5)
	The course motivated me.	5(4–5)
	I have become more interested in the field of study as a result of the course	5(4–5)

In this course, learners’ satisfaction median in the areas of " general skills of learning " in all items was 4; except in written communication skills that was 3.5(2.25-4), and regarding " specialized skills " in all items it was 4; exception the value of learning subjects for their future which was, 5(5-5) nd motivate for further learning in the field of child development that was 5(4-5) (Table 3).

**Table 3:** The learners’ satisfaction with the acquisition of general learning skills and specialized learning skills

Area	Questions	medians (quartile1,3)
General skills of learning	The course helped improve my problem-solving skills.	4(4–4)
	The course helped improve my analytical skills.	4(4–5)
	I gained valuable experience working as a team member during the course.	4(4–5)
	I have gained confidence in tackling unfamiliar problems as a result of my course.	4(3–5)
	The course helped improve my written communication skills.	3.5(2.25-4)
	The course helped improve my self-regulation skills.	4(3–4)
Specialized skills	The course stimulated my enthusiasm for further learning in the field of child development.	5(4–5)
	The course provided me with a broad overview of my knowledge in my field.	4(3.25-5)
	My perspective of my field has been broadened by this course	4.5(4–5)
	As a result of this course, I could apply principles to new situations	4.5(4–5)
	In this course, I gained greater confidence in exploring new ideas.	4.5(4–5)
	In my opinion, what I learned is valuable for my future.	5(5–5)

### Results of the second step

The codes extracted from interviews were divided into four categories; “ethical and professional commitment course teachers’ “, “being a role model in the observance of patients and their caregivers’ rights”, “course planning with a multidisciplinary approach and teamwork,“ and as well as “the use of virtual platforms to strengthen and maintain team communication between learners” (Table 4).

**Table 4:** The most important factors effective in the success of the short-term course “Developmental care for infants and toddlers” from the perspective of course learners

Open code	Sub-category	Category
Kind and compassionate	The course teachers’ ethical virtues	Ethical and professional commitment of course teachers
Humble		
Punctual		
Motivating learners		
Respecting learners		
Being available when needed		
Guiding learners		
Welcoming learners for future communication		
Friendly and intimate communication		
Having a desire to cooperate in the future		
Showing enthusiasm for teaching		
Creating a friendly atmosphere among teachers and learners		
Being a role model in teaching how to correctly communicate with patients and gain trust from them		
Teachers systematically counsel patients patiently	Playing the role of teacher committedly and formatively	
Teaching how to communicate with child patients through role modeling during the examination of children		
Being patient when announcing the final diagnosis		
Paying attention to the environmental, social, and family aspects in diagnosis		
Responding learners when they encounter challenging scenarios		
Providing tangible and practical education		
Building trust between the physician and the patient		
Teaching through observation and discussing the scenario	Being able to apply clinical teaching strategies	
Learners apply a monitoring approach during treatment and diagnosis		
Encountering children with developmental disorders in the real world		
Behaving patients humbly and respectfully	Being committed to patient rights	Being a role model in the observance of patients and their caregivers’ rights
Considering children’s physical and mental needs during treatment		
Observing ethics in diagnosis and treatment		
Being patient when examining a child suspicious of developmental problems and advising his/her parents	Gaining the patients’ and their caregivers’ trust	
Creating a fun environment for children		
Being careful and accurate in diagnosis to prevent wrong diagnoses		
Considering the economic conditions of the patient and cost-effectiveness in diagnosis and treatment		
Gathering the treatment team	Applying an interactive and formative approach to the related disciplines in education	Course planning with a multidisciplinary approach and teamwork
Having a multidisciplinary approach to diagnosis and treatment		
leading an education team that consisted of specialists from different relevant disciplines		
Forming a medical commission to examine the child from the perspective of relevant disciplines		
Using the opinions of the treatment team in providing the final diagnosis		
Having a team attitude toward the course	Implementation of a team attitude in the treatment and management of children with developmental disorders	
Teaching and deep understanding of teamwork in the diagnosis and treatment of children with developmental disorders		
Providing an objective experience of teamwork in diagnosis and treatment in the course		
Planning on the use of the areas of related disciplines		
Forming an education team consisting of various fields		
Exchanging ideas between teachers and learners to provide an appropriate treatment design	Using cyberspace to discuss problems and exchange opinions	the use of virtual platforms to strengthen and maintain team communication between learners
Using the forum to express experiences		
Using the forum to provide feedback on learners’ performance and progress		
Developing a communication network between participating pediatricians from different cities through social networks	Using social networks to continue communication after the course	
Presenting scenarios and discussing problems in the WhatsApp group to get advice from teachers after the course		
Expressing the problems learners face in their cities of residence when observing challenging scenarios through WhatsApp		
Answering learners’ questions through the forum and WhatsApp group in the leisure time of visits and training	Increasing the availability of teachers at different hours	
Daily providing feedback on the problems discusses in the WhatsApp group of the Forum		

The category " of “course teachers’ ethical and professional commitment " included three subcategories of “The course teachers’ ethical virtues”, “Playing the role of teacher committedly and formatively " as well as “the ability to apply clinical teaching strategies”.

### The course teachers’ ethical virtues

In the opinion of the participants, who were pediatricians from numerous cities in Iran, this was one of the best courses in the field of pediatrics that they have ever attended. Furthermore, Teachers’ ethical qualities, commitment, scientific expertise, and teaching skills were the most critical factors in determining the desirability of the course. Participant 2 stated, “Friendly teacher-student relationship, great enthusiasm in education, and teachers’ high knowledge were the most important features of this course.“ Participant 3 stated, " “I found the course to be a positive learning experience because the instructors were patient, spent the needed time and care in educating learners as well as examining the child patients.“ Participant 5 stated, " In addition to their compassion, kindness, and respectful behavior, the course teachers exhibited an admirable sense of responsibility.

### Playing the role of teacher committedly and formatively

Participant 8 stated,”. The course teachers were always available to respond promptly to all questions and resolve problems during all phases of visiting. " They studied the scenarios meticulously and shared their experiences with learners.“

### Their ability to apply clinical teaching strategies

Participant 3 stated, “. In addition to examining the child meticulously, the teacher demonstrated how to deal with the child with developmental disorders and how to use diagnostic tests and screening tools. " Participant 6 stated, “They had a problem-solving approach. The scenarios were meticulously examined in all dimensions, then the final diagnosis was discussed.“ Participant 7 stated, “The teacher exhibited patience in visiting the patients. The teacher exhibited patience in visiting the patients. In the following steps, the treatment team discussed different aspects of the patient’s problems, and all of the questions were carefully addressed.“ Moreover, Participant 2 stated:

” The theoretical contents were taught in person through the virtual platform designed, and the main part of the training was performed at the development center and in front of patients.“

The category of “Being a role model in the observance of patients and their caregivers’ rights” includes two subcategories: “Being committed to patient rights” and “Gaining the patients’ and their caregivers’ trust “.

### Being committed to patient rights

Participant 3 stated, “The teachers always emphasized the patients as the top priority. They attempted not to keep the parents waiting any longer. The teachers emphasized that an early diagnosis and labeling this child would be inappropriate. It is important to note that treating children in haste can lead to many problems for them and their families.“ Participants 5 also stated, “As a result of meticulously reviewing the medical history, the clinical examination, and using ASQ and Bayley scales appropriately, they avoided requesting unnecessary paraclinical tests which would have imposed unnecessary costs on the parents.“

### Gaining the patients’ and their caregivers’ trust

Participant 1 stated, “The teacher’s behaviors were a model for me. They treated patients modestly, respectfully, patiently, and kindly and tried to solve children’s and even their parents’ problems, making them easily communicate and trust the teachers.“ Participant 5 also stated, “. They provided children with an environment of fun; therefore they did not get bored too quickly, and this environment caused them to cooperate with their teachers and have a greater trust in them as a result.“

The category of “Course planning with a multidisciplinary approach and teamwork” includes two subcategories “Applying an interactive and constructive approach to the related disciplines in education “and” the implementation of a team attitude in the treatment and management of children with developmental disorders”.

### Applying an interactive and formative approach to the related disciplines in education

Participant 3 stated, " This was an interesting experience exploring different aspects of the issue and understanding the role of other disciplines in diagnosing and managing treatment using the commissions established by the developmental disabilities team.”. Interrelationships and references were found between the different disciplines. A network of interaction and cooperation contributed to the improvement of treatment.“

*The implementation of a team-based approach in the treatment and management of children with developmental disorders* Participant 5 stated, “Applying multidisciplinary approach to diagnosis, treatment, as well as the rehabilitation of children with developmental disorders changed our attitude toward the treatment and care process. In addition, it helped us recognize the importance of the treatment team in diagnosis and treatment.“

Moreover, participant 6 stated, “This course was developed to facilitate a deep understanding of the inevitable requirement of collaboration between different specialists in the final diagnosis of children with developmental disorders.”, “. Moreover, Participants 1 stated, " Among the most important strengths of this course is its ability to improve teamwork and provide a deep understanding of how it contributes to therapeutic progress, i.e., a team including a pediatrician, a developmental-behavioral pediatric, a neurologist, a pediatric psychiatrist, a clinical psychologist, an occupational therapist, a speech therapist, a therapist, a Psychometrist, an adult psychiatrist, as well as a family counselor.“

The category of “the use of virtual platforms to strengthen and maintain team communication among learners” includes three subcategories “Using cyberspace to discuss problems and exchange opinions”, “Using social networks to maintain communication after the course”, and “Increasing the availability of teachers at different hours”.

### Using cyberspace to discuss problems and exchange opinions

Participants 5 stated, “We discussed patients’ problems and raised our questions through social networks. One of the course facilities was a virtual training system in the comprehensive development center which made it possible to discuss our cases, transfer our experiences, and exchange our opinions.“

### Using social networks to maintain communication after the course

Participant 4 stated, “Participating in discussions in the virtual group can lead to the maintenance of communication between participants and teachers. In addition, Participant 2 stated: “Using virtual networks provided the friendly atmosphere. Colleagues from different cities could communicate well. Each other’s experiences were shared. Furthermore, the networks provided a platform to communicate interchangeably with each other and the teachers in the future.“

### Increasing the availability of teachers at different hours

Participant 5 stated, “Teachers daily responded to emails and participated in the discussions. Given that we were from different cities, the teachers easily responded to the cases we encountered at work.“ Participant 4 also stated, " Our teachers diligently investigated our issues at the end of the day, when we were usually done with our daily work, in the WhatsApp group already created.“

## Discussion

The current study aimed to evaluate the first short-term supplementary course, on a multidisciplinary “Developmental Care for Infants and Toddlers,“ and investigate its effectiveness from the perspectives of its graduates. This course was developed based on the needs of the learners and the graduates of this course, as focal points in the area of infant and toddler development among pediatric specialists in addition, they were responsible for providing services at their workplaces in different cities of Iran. According to the results of the quantitative part, learners were most satisfied with the “acquisition of specialized skills” and " learners’s motivation” to continue their education and acquire knowledge in this field, indicating the success of this course in achieving its ultimate goal. In this course, it was attempted to create a cooperative and friendly atmosphere among learners. In a study conducted by Hazrati H et al. (2021), enculturating teamwork in health care is the first step towards improving multidisciplinary education.

Teachers of the courses will make other teachers available to help learners solve their diagnostic and treatment problems as well as provide consultation to them until the full establishment of their Child Development Centers in their respective cities has been accomplished. Various studies have also introduced effective communication skills such as ethical, behavioral, and emotional interaction with learners, guidance and counseling, confidence building, flexibility, and motivation which are listed among the most important factors making clinical education effective [9–11]. In educational design, the use of appropriate clinical education strategies led to the learners’ satisfaction with the course [12]. This course mainly focused on observing the performance of experienced and specialized physicians in the field of child development, conducting independent visits and presenting them to teachers, discussing the disorder, and making a joint decision on the plans needed in TCDC and Tabriz Children’s Hospital. The next step included the learners’ activities and visiting their clients with developmental problems in their cities along with teachers’ feedback provided on their performance. Various studies have mentioned the acquisition of competencies through mentorship training provided by experienced teachers and behavioral modeling of them as an experience of successful clinical training [13, 14]. According to Altshuler et al. (2015), it has helpful for junior teachers to observe their master teachers’ providing medical history, and clinical reasoning skills. In addition, some participants have stated that the training helped them recognize their shortcomings in clinical education. Moreover, it has been found effective for junior teachers to focus on the teaching skills of their master teachers’. They include the ways of involving learners in the clinical reasoning process, assessing students’ clinical reasoning skills in physical examination, as well as presenting a scenario, that can involve the learners as a team member in order to improve their providing medical history and clinical reasoning skills [15]. In their study, Finn et al. have defined the observation of experienced clinical teachers and their feedback to train junior clinical teachers as appropriate techniques [16].

The current multidisciplinary course was designed due to its interdisciplinary nature. Due to their objective experience of multidisciplinary education, the participants have described this course as the best and first experience of using different disciplines in pediatric treatment management. Based on their experience, the course has provided a platform for them to closely understand the role of related disciplines in treating patients with developmental disorders. Nowadays the complexity of patient care needs and the prevention of diseases make it impossible for anyone specialty to meet the needs of patients on its own, and different relevant disciplines must manage the treatment [11]. Therefore, Multidisciplinary approaches are becoming more prevalent in education and treatment globally. In accordance with the American Medical Association, patients receive safe and high-quality medical care when healthcare professionals work effectively together through formative communication, mutual understanding of the roles, respect as well as trust. [17]. Therefore, eliminating mental stereotypes about specialization and superiority of medical sciences over other disciplines will reduce tensions and conflicts between different health care professions [18, 19], In addition it will provide an opportunity to achieve a standard value system manage and treat patients [16, 17] properly. According to the analysis of learners’ experiences, the personality characteristics of the teachers and their ethical and professional commitment contributed to the continuation of the cooperation between learners and teachers. As a result of these items, learners have been provided with a respectful, friendly, and incentive-rich atmosphere, and teachers have been teaching ethical and professional communication skills by role modeling. Various studies have indicated that when providing medical care and serving as a teacher, learners can be explicitly encouraged and institutionalized to practice human virtues such as altruism, kindness, and empathy [20, 21]. Sutkin et al. (2008) have classified the most important factors influencing clinical education into three categories: practicing, teaching, and individual characteristics.

As a physician, clinical teachers should possess medical/clinical knowledge, have an enthusiasm for medicine, emphasize the physician-patient relationship, demonstrate professionalism, be scholarly, value teamwork, have leadership, and possess collegial and management skills [10]. As a teacher, he/she should have positive communication skills, support students, demonstrate enthusiasm for teaching, be available to students, and provide feedback and formative assessment. As a human, he/she should be personable, compassionate, kind, honest, knowledgeable, intelligent, cheerful, responsible, respectful of others, and able to balance professional and personal responsibilities [22, 16]. In this course, due to the participants residing in different cities, virtual platforms were used to facilitate communication between the students and the teachers, which led to more interaction.

According to learners, using virtual learning platforms has provided the possibility of effective distant communication in the future. During this course, a team of specialists has been assembled from various parts of Iran. Virtual learning platforms have been found to have numerous advantages according to a number of studies. For example, Shah Goli et al. have identified the most advantages of virtual learning platforms as follows: attention to audiences’ needs, easy access to various resources, the possibility of recording activities and programs, continuous monitoring of academic progress, as well as providing counseling to learners [23].

## Conclusion

In this course, interdisciplinary approaches were applied for the first time in an interprofessional program. For the Iranian medical education system to achieve its educational and therapeutic goals, it must move away from specialization and connect all related specialties and disciplines. Based on the experiences of course participants, it is evident that the course managers’ sense of responsibility and ability to communicate effectively with learners and consultants from other disciplines can play a critical role in the success of multidisciplinary training courses. This course can serve as a model for the design of courses in other disciplines and clinical settings.

## Data Availability

The data supporting this study’s findings are available from “National Agency for Strategic Research in Medical Education, Tehran. Iran.” However, restriction applies to the availability of this data, which were used under license for the current study; therefore, they are not publicly available. It is, however, possible to obtain data from the corresponding author (Hakimeh Hazrati, Email: hakimeh.hazrati@gmail.com) upon reasonable request and with the permission of “National Agency for Strategic Research in Medical Education, Tehran”.
